# Cases in Practice

**Published:** 1889-03

**Authors:** T. L. Lallerstedt

**Affiliations:** Ga.


### Southern Medical Record.

A MONTHLY JOURNAL OF PRACTICAL MEDICINE.

☞ All communications must be addressed to R. C. Word, Managing Editor.

"*Quicquid Præcipies Esto Brevis.*"

VOL. XIX ATLANTA, GA., MARCH, 1889. NO. 3

### ORIGINAL AND SELECTED ARTICLES.

### CASES IN PRACTICE.

By T. L. Lallerstedt, M. D., of Ga.

*Shoulder Presentation.*—The few words I shall write on this subject are suggested by reading report of Dr. Beauchamp's trouble in the Dec. No. of the Record.

I have had two shoulder cases in my own practice, and assisted a brother practitioner in another case. In my first case and in that of the brother practitioner there was considerable trouble. The last case I managed alone, with comparative ease. On Dec. 12th, 1887, I was called to attend Mrs. W. in confinement. On examination I found that the membranes had been ruptured for some time, and an arm was protruding. Having read an article recommending the placing of the patient in the knee chest position in such cases I so placed the patient and promptly succeeded in putting back the arm, and correcting the presentation. The child was soon delivered in the natural way. It was dead, but the mother had a good recovery.

*Hernia in a child.* Since the beginning of the present year I was called to see a two year old boy with strangulated hernia. His father had been in the habit of putting it back, but on this occasion had failed after several hours of effort. I made an attempt to reduce it in the usual way, for a short while without success, when I thought of the plan of lowering the head and thus securing the advantage of gravity. The plan succeeded in less time than I have taken to write the fact.

*Bronchitis.* For the last year I eave had very good success in treating bronchitis with iodide of potassium in doses of 16 to 24 grs. a day.

I am now treating two patients, one aged 14 years, the other 70. My way of using it is to put an ounce of the iodide in 4 ounces of simple syrup—give 10 to 20 drops every four hours, according to age, and the quantity I desire them to have, any child will take it in this shape, when mixed with a little water.

*Dyspepsia.* For the last four years I have found best success in dyspepsia with the following prescription:

| | | |
|---|---|---|
| ℞ | Pulv. opii, . . . . . . | grs. iv. |
| | Sub. nit. bismuth, . . . . . | grs. xl. |
| | Cinchonidea Sulph. . . . . | grs. xxxii.. |

Put into capsules No. xvi. (P. D. & Co. No. 1). Give one capsule before each meal. After meals give 10 drops tinct. nux vomica to an adult, to encourage peristalsis, and at bed time give a capsule of the following formula to keep bowels open.

| | | |
|---|---|---|
| ℞ | Aloes pulv. | |
| | Ferri sulph. . . . . . . | ää ℈ii. |
| | Oil sassafras . . . . . | vi drops. |

Put into 20 capsules, unless the bowels become too loose. [The quantity of the ferri sulph. and the tinct. of nux seems too large.—ED. OF RECORD.]

